# Prenatal diagnosis of intellectual disability, autosomal dominant 29 with a nonsense pathogenic variant in *SETBP1*: a case report and literature review

**DOI:** 10.3389/fgene.2025.1463485

**Published:** 2025-03-12

**Authors:** Zhuo Wei, Liying Yao, Lei Zhang, Shanshan Li, Meiyi Xu, Dan Wu, Wen Li, Ying Chang

**Affiliations:** ^1^ Tianjin Institute of Obstetrics and Gynecology, Tianjin Central Hospital of Obstetrics and Gynecology, Tianjin, China; ^2^ Tianjin Key Laboratory of Human Development and Reproductive Regulation, Nankai University Affiliated Maternity Hospital, Tianjin, China; ^3^ Prenatal Diagnosis Center, Nankai University Affiliated Maternity Hospital, Tianjin, China

**Keywords:** MRD29, *SETBP1*, prenatal diagnosis, WES, cortical abnormalities

## Abstract

**Introduction:**

Intellectual disability, autosomal dominant 29 is a rare disorder resulting from pathogenic variants of *SETBP1* gene with no specific mutation hotspot identified. Systematic descriptions of new cases are crucial for understanding the genotypic and phenotypic spectrums of the disease.

**Case presentation:**

A pregnant woman was referred to the prenatal diagnosis center at our hospital because she has an intellectual disability and has previously given birth to a child with intellectual disabilities. Karyotype, CNV-seq and whole-exome sequencing (WES) were employed to investigate the potential genetic issues in the family. The *SETBP1* NM_015559.2: c.2425C>T (p.Gln809*) nonsense variant was found in the proband and mother, who were diagnosed with MRD29. Amniocentesis and genetic analysis (CNV-seq and sanger sequencing for mutation site) were performed as fetal cortical abnormalities and subependymal cystic area presented by ultrasonic examination at 25 + 5 gestational weeks. The genetic analysis confirmed the *SETBP1* c.2425C>T (p.Gln809*) nonsense mutation in the fetus. The parents terminated the pregnancy at 30 + 4 gestational weeks.

**Conclusion:**

The *SETBP1* NM_015559.2: c.2425C>T (p.Gln809*) nonsense variant is pathogenic and *SETBP1* haploinsufficiency may be associated with fatal cortical abnormalities. More prenatal clinical data is helpful for a better productive decision making and patient management.

## 1 Introduction

Intellectual disability, autosomal dominant 29 (MRD29, MIM #616078) is a rare disorder that commonly associated with speech impairment, mild motor developmental delay and intellectual disability as reported in small case series. Additionally, hypotonia, vision impairment, concentration deficits, and hyperactivity have been documented in several cases. Prior to Jansen et al.’s delineation of the clinical spectrum of MRD29 among 34 individuals in 2021, there were no systematic descriptions of the disorder’s phenotypic and genotypic spectrums (Jansen et al., 2021). Subsequently, Morgan et al. emphasized the centrality of speech and language deficits among 31 individuals (Morgan et al., 2021). Nevertheless, the underlying mechanisms remain unclear.

With the advancement of next-generation sequencing (NGS), SET binding protein 1(*SETBP1*) has been identified as the disease-causing gene for MRD29. *SETBP1* gene is located at 18q12.3 and encodes a protein with molecular mass of ∼170 kDa in most tissues. The SETBP1 protein possesses multiple functional domains, including a SET-binding region, an oncoprotein SKI homologous region, three bipartite NLS (nuclear localization signal) motifs, three AT hook domains, six PEST sequences, three sequential proline-rich repeats, four KxKHKxK, eight LSxxL and ten PxxPS repeated sentences (Minakuchi et al., 2001). The SKI-homology domain shares homology with the nuclear oncoprotein SKI and contains a degron motif that is recognized by the proteasome for protein degradation (Minakuchi et al., 2001).

MRD29 is believed associated with heterozygous gene deletion or loss-of-function (LoF) variants of *SETBP1*, without clear mutation hotspots (Jansen et al., 2021). In contrast, gain-of-function mutations in the SKI domain lead to the more sever Schinzel-Giedion syndrome (SGS, OMIM ID: 269150) characterized by recognizable facial characteristics, severe intellectual disability, and various congenital anomalies (Hoischen et al., 2010). These observations indicate a dose-dependent effect of *SETBP1*. However, the underlying mechanism of how altered SETBP1 protein dosage affects brain development remains elusive. Research utilizing human embryonic stem cells (hESCs) has demonstrated that *SETBP1* deficiency affects forebrain progenitor expansion and neurogenic differentiation (Cardo et al., 2023). Nevertheless, few cases have reported abnormal brain MRI findings, implying that there may be issues with the timing of brain development detection.

In this report, clinical and molecular findings in a Chinese family with MRD29 are presented. Whole-exome sequencing (WES) analysis identified a nonsense variant. To discuss the prenatal diagnosis of MRD29 disease and improve understanding of the disease, previously reported cases were reviewed.

## 2 Case presentation

A 29-year-old pregnant woman, gravida 3, para 1, was referred to Tianjin Central Hospital of Obstetrics and Gynecology due to intellectual disability and a history of intellectual disability childbirth at 19+5 weeks of gestation. The pregnant woman exhibited intellectual disability, delayed language development, and could not use complete sentences before the age of 14 years old. She and her partner were un-related, and no disorder was reported about her partner. Their first child, a 6-year-old son (proband), presented with intellectual disability (Wechsler Intelligence Scale for Children-IQ test score of 52) and an inability to use complete sentences.

**FIGURE 1:**
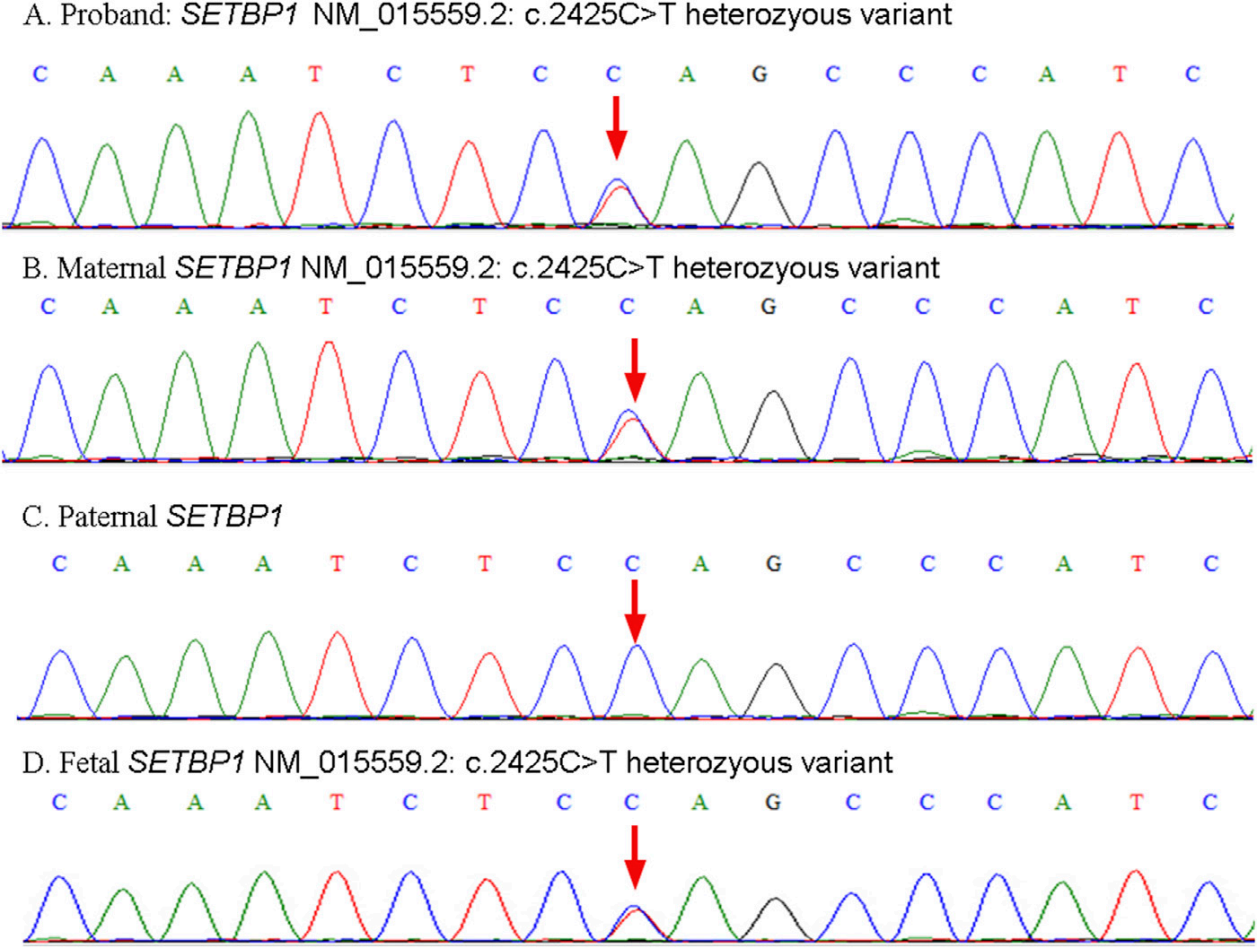
Schematic representation of validation results by Sanger sequencing. The heterozyous variants presented in the proband **(A)**, mother **(B)** and fetus **(D)**. The wild type SETBP1 presented in the father **(C)**.

Peripheral blood samples of the parents and proband were collected at 20 weeks gestational age for karyotype analysis and chromosome copy number variation sequencing (CNV-seq) initially. As no abnormality was detected but nonnegligible genetic predisposition, trio-exome sequencing was then employed. Written informed consent was obtained from patients clarifying the benefits and risks of clinical whole-exome sequencing testing. As expected, the mother and proband were found to have a heterozygous *SETBP1* c.2425C>T (p.Gln809*) nonsense mutation (Figures 1A, B). While, no pathogenic variant of the SETBP1 gene was detected in the father (Figure 1C). Considering the clinical features presented and potential genetic mechanism, the proband and mother was diagnosed with MRD29.

**FIGURE 2:**
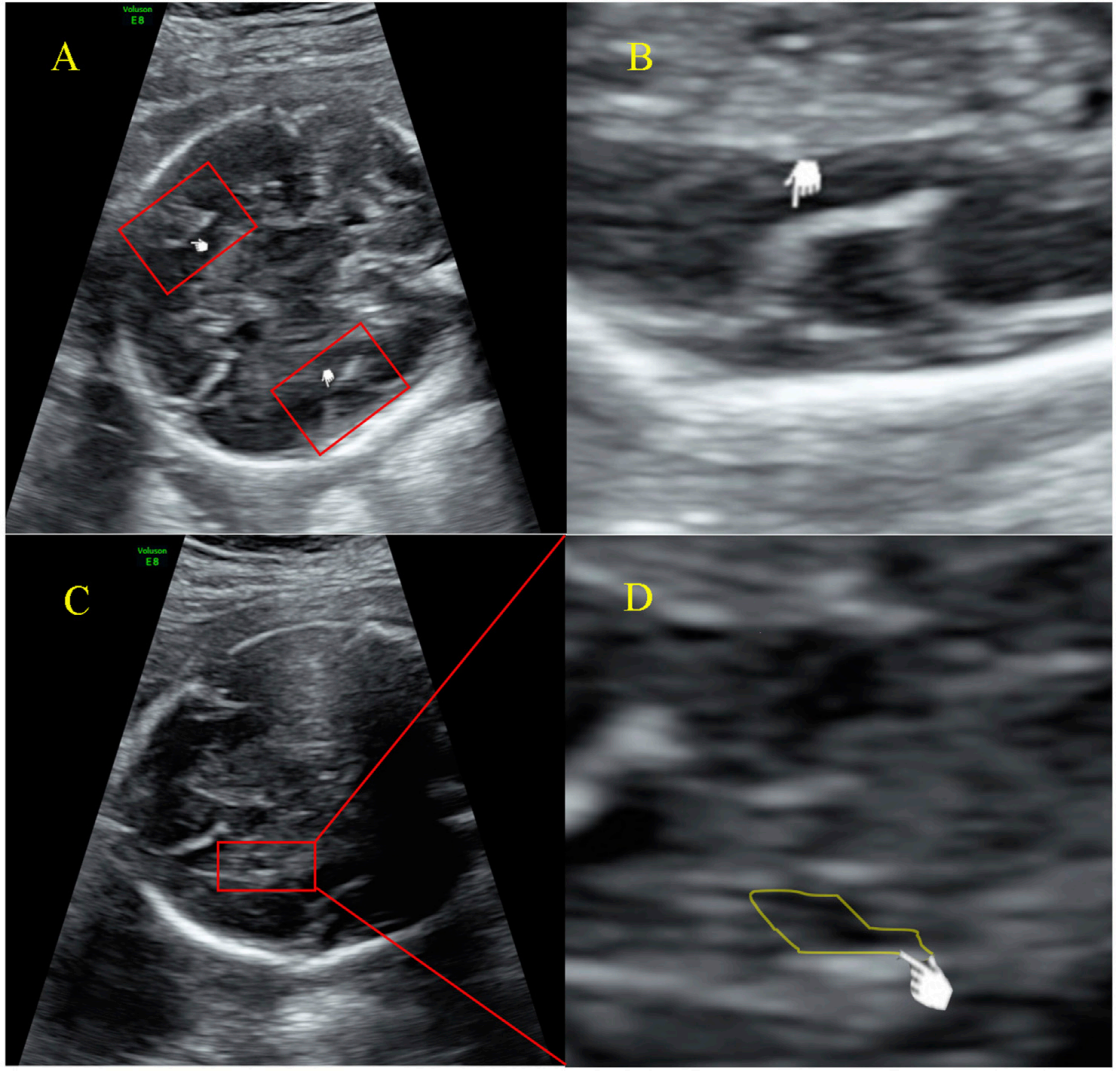
Ultrasound examination image of the fetal **(A)** Malformation of cortical development indicated by an increased Sylvian fissure angle; **(B)** Measurement of the Sylvian fissure angle; **(C)** Subependymal cyst; **(D)** Magnified image of the indicated cystic area, presented by ultrasonic examination at 25+5 weeks of gestation.

At 25+5 weeks of gestation, fetal cortical abnormalities and subependymal cystic area were detected by ultrasonic examination (Figure 2). Subsequently, amniocentesis was performed at 26+2 weeks of gestation for genetic analysis (CNV-seq and Sanger sequencing). A heterozygous SETBP1 c.2425C>T (p.Gln809*) nonsense mutation was detected (Figure 1D), and the fetus was subsequently diagnosed with MRD29 prenatally. The parents chose to terminate the pregnancy at 30+4 weeks of gestation and declined a post-mortem examination of the fetus. The patient’s general condition was good at discharge.

**FIGURE 3:**
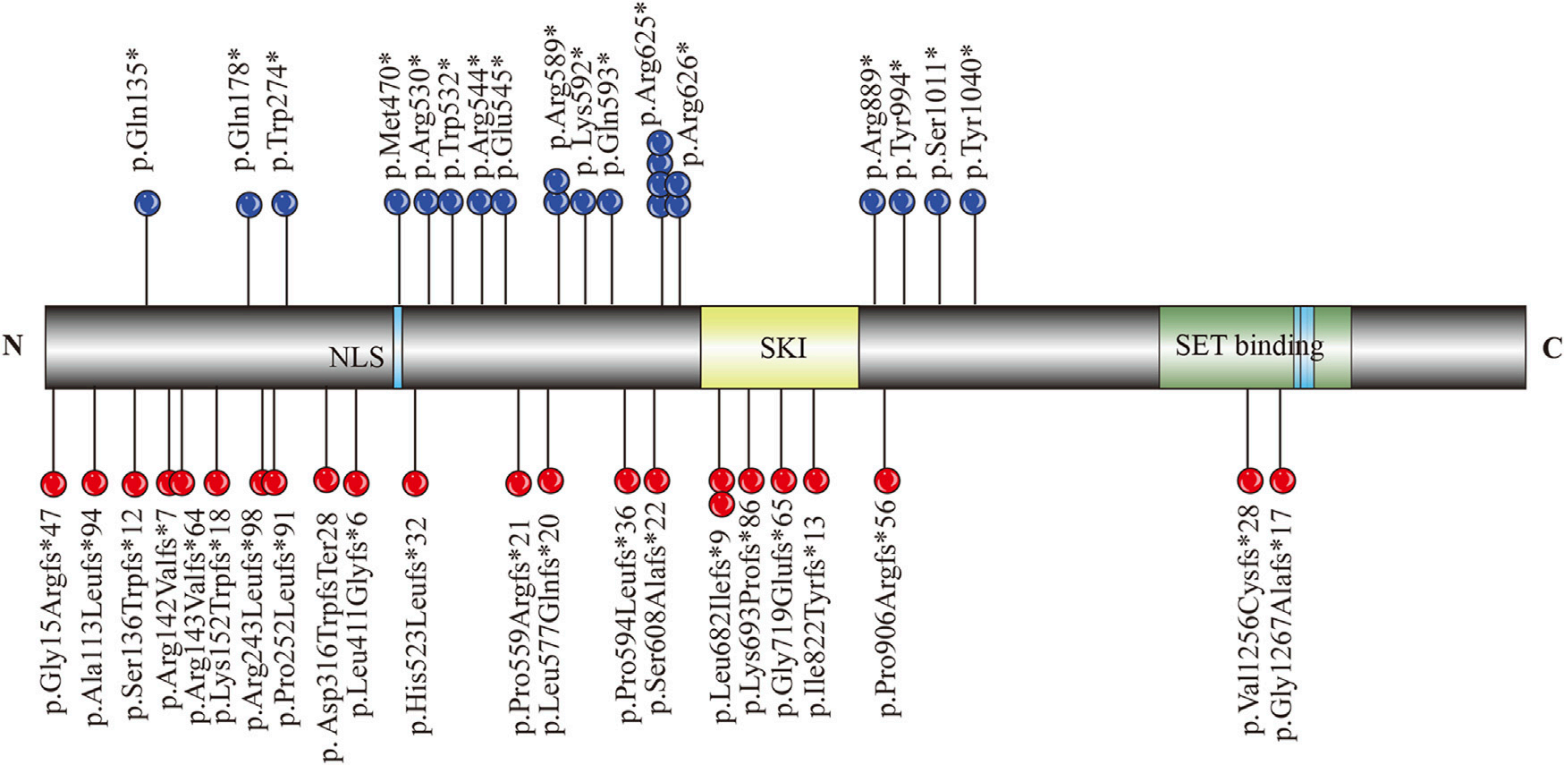
Schematic diagram of SETBP1 functional domains and variants identified in relation to MRD29.

To summarize the clinical phenotype of MRD29 disorder, “MRD29 and SETBP1” were used as the formula for literature retrieval in the PubMed database. All variants and their positions are summarized in Figure 3. The clinical spectrum of individuals, including prenatal and brain MRI findings, is systematically outlined in Supplementary Table 1.

## 3 Discussion and conclusion

We report a *SETBP1* c.2425C>T variant here, and the mutation results in the 809th amino acid, glutamine, in the protein SKI domain replaced by a stop codon and causes termination of the SETBP1 protein. This is the first time a fetus with SETBP1 haploinsufficiency has been reported. Given the limited number of MRD29 cases reported to date, it is critical to focus on the phenotypic features of individuals with different variants. We systematically evaluated the phenotypes in 59 individuals reported to date: speech delay was reported in almost all cases evaluated (55/56, 98.21%); motor development delay (51/57, 89.47%) and intellectual disability (49/51, 96.08%) were also reported in almost all cases; 22 out of 42 (52.38%) cases had a history of feeding difficulties; 19 out of 36 (52.78%) cases were reported with vision impairments, including hypermetropia (9 cases), astigmatism (4 cases), strabismus (6 cases), myopia (3 cases), amblyopia (1 case), color blindness (1 case), and lack of binocular vision (1 case); hypotonia (19/30, 63.33%) and attention/concentration deficit (29/41, 70.73%) were also commonly reported, as shown in Supplementary Table 1 (Jansen et al., 2021; Morgan et al., 2021; Marseglia et al., 2012; Hamdan et al., 2014; Miolo et al., 2024; Kaspi et al., 2023; Vrkic Boban et al., 2022; Wang et al., 2023; Zhou et al., 2022; Rauch et al., 2012; Filges et al., 2011; Coe et al., 2014; Eising et al., 2019; Hildebrand et al., 2020; Alesi et al., 2024). The clinical findings in this family align with current knowledge on the spectrum of MRD29, including speech problems and intellectual disability.

We have reported for the first time the delayed development of the Sylvian fissure in the fetus as well as subependymal cysts. As we illustrated in Figures 2A, B, the development of Sylvian fissure was delayed according to works conducted by Chen et al. (2017) and Pooh et al. (2019). These works summarized the changing appearance on prenatal ultrasound of the sylvian fissure and determined sylvian fissure changes as important part of fetal cortical development. Interestingly, most individuals were previously reported to have normal brain MRI scans, with the exception of three cases under 4 years old who were identified with delayed myelination (Jansen et al., 2021; Morgan et al., 2021; Hamdan et al., 2014; Filges et al., 2011; Coe et al., 2014). In line with these reports, the mother and proband also presented normal MRI scans in this Chinese family. This finding underscores the need for further investigation into the role of *SETBP1* in neurological phenotypes during early brain development, as well as its potential association with speech and language disorders at an early stage of life. However, our understanding of the prenatal characteristics of the MRD29 disorder remains limited, with only a few reported cases involving amniotic fluid abnormalities, fetal heart arrhythmia, fetal heart bradycardia, dysmaturity, hypotonia, fetal distress, and the presence of a single uterine artery. Further research is warranted to elucidate the full prenatal profile of this disorder and to establish a correlation analysis between prenatal and postnatal phenotypes, enabling personalized management strategies for patients.

Mechanistically, Lucia F. et al. have revealed that *SETBP1*-deficiency affects forebrain progenitor expansion and neurogenic differentiation by CRISPR/Cas9 genome editing hESC lines (Cardo et al., 2023). However, the precise role of SETBP1 in aggravating brain pathology remains unclear. Specifically, the cerebral cortex, particularly the posterior regions surrounding the Sylvian fissure, is crucial for regulating speech and language functions. Recently, Cabet S. et al. found that a prenatal lack of opercularization of the Sylvian fissure, without any other extracranial anomalies, is associated with speech delay (Cabet et al., 2024). Given our observation of delayed development of the Sylvian fissure in certain cases, we hypothesize that *SETBP1* plays a role in the development of this fissure, which in turn regulates language and speech abilities. To clearly explore the influence of *SETBP1* mutation on the development of the Sylvian fissure, animal experiments should be conducted. It is also important to note that more high-quality cases describing prenatal findings are needed, given the potential for significant heterogeneity in the manifestation of SETBP1 disorders.

## Data Availability

The original contributions presented in the study are publicly available. This data can be found here: ClinVar repository, accession number SCV005442721, https://www.ncbi.nlm.nih.gov/clinvar/variation/807682/?oq=SCV005442721&m=NM_015559.3(SETBP1):c.2425C%3ET%20(p.Gln809Ter).

## Supplementary material

SUPPLEMENTARY TABLE 1 SETBP1 variants and clinical characteristic related to MRD29.
